# Novel Porous Brain Electrodes for Augmented Local Field Potential Signal Detection

**DOI:** 10.3390/ma12030542

**Published:** 2019-02-12

**Authors:** Sung Hyun Lee, Kyeong-Seok Lee, Saurav Sorcar, Abdul Razzaq, Maan-Gee Lee, Su-Il In

**Affiliations:** 1 Department of Energy Science & Engineering, DGIST, 333 Techno Jungang-daero, Hyeonpung-myeon, Dalseong-gun, Daegu 42988, Korea; mattlee@dgist.ac.kr (S.H.L.); dwm2002@dgist.ac.kr (K.-S.L.); sorcar@dgist.ac.kr (S.S.); 2 Department of Chemical Engineering, COMSATS University Islamabad, Lahore Campus, 1.5 KM Defence Road, Off Raiwind Road, Lahore 54000, Pakistan; abdulrazzaq@cuilahore.edu.pk; 3 Department of Pharmacology, School of Medicine, Brain Science and Engineering Institute, Kyungpook National University, Chilgokjuangangdae-ro 136-gil 90, Buk-gu, Daegu 41405, Korea; mglee@knu.ac.kr

**Keywords:** brain, local field potential, neural networks, porous neural electrodes

## Abstract

Intracerebral local field potential (LFP) measurements are commonly used to monitor brain activity, providing insight into the flow of information across neural networks. Herein we describe synthesis and application of a neural electrode possessing a nano/micro-scale porous surface topology for improved LFP measurement. Compared with conventional brain electrodes, the porous electrodes demonstrate higher measured amplitudes with lower noise levels.

## 1. Introduction

The brain contains numerous cells and neurons that convey information in the form of action potentials [1,2,3]. Electrical signals are useful to understand neuron function(s), and the functional connectivity between brain regions [4,5]. Measurement of local field potential (LFP) [6,7,8], through the use of neural electrodes, is a common method for monitoring electrical brain signals. With respect to neural electrode design low brain signal intensity mandates use of low impedance electrodes, and smaller electrodes are preferable to minimize tissue damage. However, there has historically been a trade-off between electrode size and the impedance, with an inverse relationship between the two properties [9,10,11].

Several types of electrodes have been implanted for the measurement of LFP to study the central nervous system (CNS). For clinical settings, particularly, silicon based electrodes/probes were most commonly used due to many significant advances corresponding to deep brain stimulation and neural recordings [12,13,14,15,16,17]. The Michigan probes [18,19,20,21,22] and the Utah array [23,24] are well known for silicon based probes that have been proved to be excellent due to a wide range of their applications. The Utah array electrode is microelectromechanical system (MEMS) with an approval for human trials is based on 10 × 10 array of silicon needles with a single electrode on each needle tip [14,23,25]. Wise et al., developed silicon-based electrode arrays at the cellular level and in particular studied for an increased number of recording sites [26]. Campbell et al., developed a “three-dimensional” electrode array of 100 penetrating intracortical electrodes with the goal of chronic intracortical stimulation. The as developed conical shaped geometry of array is well suited for chronic implantation in cortex. The low and high impedance values were recorded along the needle and between the electrodes respectively, which were well suited for stimulation of cortical tissue. The fabrication process of both Michigan probes and Utah array were based on the wet or dry etching of silicon [27,28,29]. Furthermore, silicon based neural probes with new fabrication technique assembled in multifunctional i.e., two-dimensional (2D) and three-dimensional (3D) microprobes for neural recording were developed [30]. Here, the thickness of probe was adjusted during the fabrication which helps to achieve a balance between minimal tissue damage during the implantation and probe stiffness. Along with silica based materials few biocompatible polymers that includes SU-8 [31], polyimide [32] and parylene (C) [33,34] and neural prosthetic devices (NPDs) [35] were employed for neural sensors.

Herein, we report a novel neural electrode possessing a nano/micro-scale porous topology for LFP application. Within the auspices of a systematic study using Sprague-Dawley rats the performance of the porous neural electrodes (PNEs) are compared to conventional neural electrodes. We find the PNEs show significantly improved LFP signal quality, enabling a significant advance in measurement technology.

## 2. Materials and Reagents

Stainless steel needles (6 cm length and 0.3 mm diameter, Dong Bang Medical Inc., Seongnam-si, Korea) were used as conventional neural electrodes (CNE); porous neural electrodes were fabricated by electrochemical anodization of CNEs. Ammonium fluoride salt (NH_4_F, 98.0%), for electrolyte preparation was obtained from Daejung Chemicals and Metals Co., Ltd., Shieung-si, Korea, and ethylene glycol (Extra pure, >99.0%) as an electrolyte solvent was obtained from Alfa Aesar, Incheon, Korea. All the materials and reagents were used as received without any further modification.

### 2.1. Fabrication of Porous Neural Electrode (PNE)

**Figure 1:**
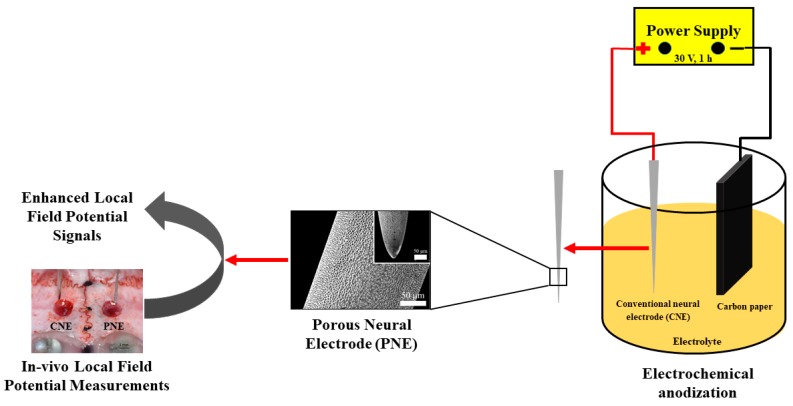
Schematic flow diagram depicting the synthesis and application of PNE for enhanced LFP signal detection (PNE, porous neural electrode; LFP, local field potential).

PNEs were fabricated as earlier described [36]. Briefly, a CNE was washed sequentially in acetone, ethanol and deionized (DI) water via sonication (5 min each) to remove any potential surface contaminants. The cleaned CNE were electrochemically anodized using a two-electrode cell, with CNE as a working electrode (anode) and carbon paper as the counter electrode (cathode). The electrolyte used was prepared by dissolving 0.3 wt.% NH_4_F in ethylene glycol with 2.0 vol.% DI water. The anodization was performed at 30 V for 1 h. The resulting PNEs were then rinsed with acetone, ethanol, and DI water then dried at ambient temperature under a stream of nitrogen gas. A schematic depiction of PNE synthesis and its in-vivo test is given in Figure 1.

### 2.2. Characterization of Neural Electrodes

Surface morphologies were studied using a Field Emission Scanning Electron Microscope (FE-SEM, Hitachi S-4800, operating at 3 kV, Hitachi High Technologies, Tokyo, Japan). Electrode electrical properties were investigated using electrochemical impedance spectroscopy, performed using a potentiostat (Bio-Logics SAS, Model VSP-1158, Bio-Logic, Seyssinet-Pariset, France) with a three-electrode workstation; Measurements were conducted in the frequency range of 200 kHz–50 mHz; fresh as synthesized neural electrodes served as the working electrode, a Pt wire was used as the counter electrode, and an Ag/AgCl reference electrode. All three electrodes were dipped in saline solution (electrolyte). The electrolyte solution consists of 0.9 g NaCl in 100 mL DI water purchased from JW-Pharma, Dangjin-si, Korea and used without further purification.

### 2.3. Animal Preparation

**Figure 2:**
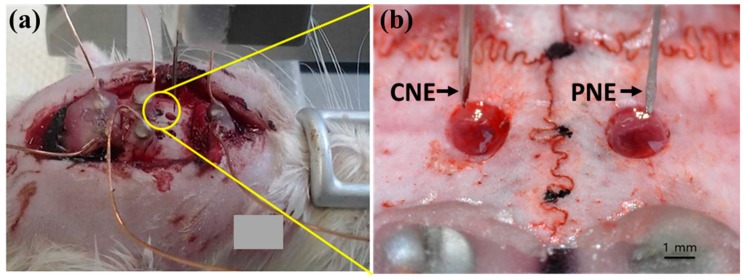
(**a**) Rat being fixed in a stereotaxic apparatus mounted with a vertical micromanipulator, and (**b**) magnified image showing the region circled in yellow where holes are made on the skull in the diameter of approximately 2 mm with CNE and PNE stationed above (conventional neural electrodes, CNE).

Three Sprague-Dawley rats (weight 300–350 g) were used in this study. The rats were anesthetized with urethane (1.5 mg/kg, i.p) and fixed in a stereotaxic instrument. The scalp of each rat was regionally anesthetized with 1% of lidocaine subcutaneously injection, cut along the midline, and the periosteum was removed. Two, 2 mm diameter holes were made on the skull above the motor cortex or parietal association cortex (Figure 2a) and the dura was cut to allow easy insertion of the electrodes. This experiment was approved by the Kyungpook National University Institutional Animal Care and Use Committee (No. KNU 2016–0082-1), and was performed according to Guide for the Care and Use of Laboratory Animals (National Institute of Health, 1996).

### 2.4. Local Field Potential (LFP) Analysis

Both electrodes were fixed on micromanipulator arm attached to the stereotaxic instrument. The electrodes were connected to assigned amplifier channels (Figure 2b): left pin electrode to channel 1; right pin electrode to channel 2; and a screw electrode to reference input of the amplifier (AM Systems, Model-1600, A-M Systems, Carlsborg, WA, USA). Signals were amplified, and then sampled at 200 Hz with an analog-to-digital converter (Model-DigiData 1320, Axon Instruments, Inc., Foster City, CA, USA). Signals were recorded from the PNE and CNE as they were vertically descended from the cortex surface in 1 mm increments up to 5 mm depth. The LFP and noise signals were recorded for 10 min at each depth. Since environmental factors, including anesthesia, can influence LFP signals [2], a pair of porous (PNE) and non-porous electrodes (CNE) were used at the same time for each experiment. A total of 16 electrodes were used in 3 rats. A pair of electrodes (from one CNE and one PNE) was used for recording at one time. In two rats, each was recorded one time in the motor cortex and one time in the parietal cortex (using a total of 8 electrodes). In one rat, two times in the motor cortex and two times in the parietal cortex (using a total of eight electrodes). At each depth power ratios over two different frequency ranges (1 Hz–49 Hz, 59 Hz–61 Hz) were calculated by power spectrum analysis using signals from the last five minutes of each ten-minute recording. In this study the power ratio from 1 Hz to 49 Hz was considered as a valid LFP signal [37,38], and the power ratio from 59 Hz to 61 Hz considered as noise [37,38]. Analysis of Variance (ANOVA) was used to compare CNE and PNE measurements; Tukey’s test was conducted and *p* < 0.05 considered as a significant difference. One of the two screw electrodes over the cerebellum was used as a counter electrode (reference electrode) of the pin electrode in vivo.

## 3. Results and Discussions

**Figure 3:**
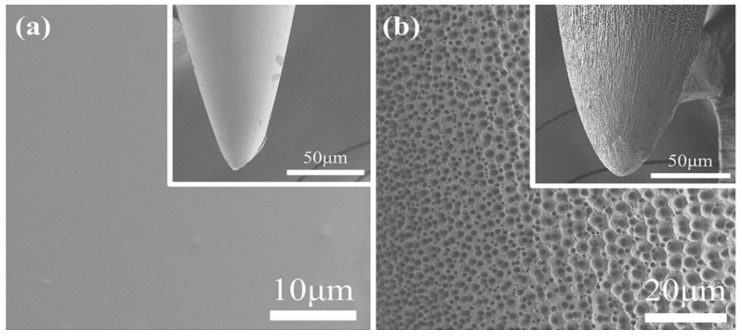
Surface Field Emission Scanning Electron Microscope (FE-SEM) images of (**a**) CNE and (**b**) PNE.

Figure 3a is a surface image of an illustrative CNE, and Figure 3b that of a PNE. It can be clearly observed that the CNE (Figure 3a) possesses a smooth non-porous surface, however after anodization at 30 V for 1h, the fabricated PNE shows a porous surface morphology with pores of a nano-micro nature (Figure 3b). We have performed FE-SEM studies of additional two sets of PNE and CNE. The results are given in supplementary information (Figures S1 and S2) and one can see PNE have pore size of 1.00 μm–1.30 μm. The formation of nano-micro pores over the PNE can be attributed to electrochemical etching due to the NH_4_F [36]. In an alkaline medium, etching begins by pitting the surface of a CNE, which proceeds into the interior to form micro/nano surfaces. The surface topology of PNE provides a higher interfacial surface area, resulting in improved contact with an encompassing medium and enhanced LFP signal transfer. Elemental analysis of PNE and CNE are performed using energy dispersive spectroscopy (EDS). The data (Tables S1 and S2, supplementary information), confirms no major loss in chemical species in neural electrodes takes place. Therefore, we believe that electrochemical anodization technique is a pertinent approach for fabricating PNEs. Biocompatibility tests like physical properties and animal model studies of PNEs were conducted in our previous work [39]. The results have proven that PNEs are safe to be used for therapeutic practices.

**Figure 4:**
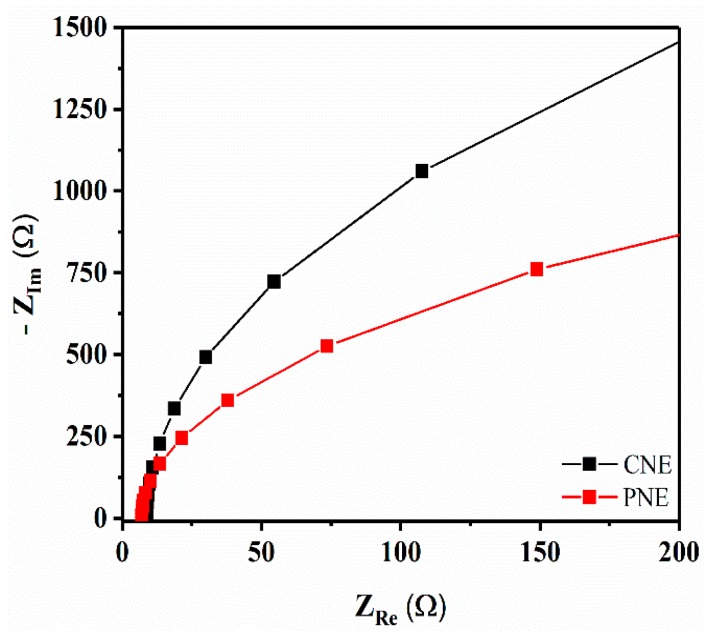
Fitted Nyquist plots from electrical impedance measurements of CNE and PNE (number of samples tested = one, N = 1; frequency 200 kHz–50 mHz and amplitude = 10 mV; measurements performed using saline electrolyte solution of 0.9 g NaCl in 100 mL deionized (DI) water).

Fitted Nyquist plots for fresh CNE and PNE are shown in Figure 4. It can be observed that both CNE and PNE exhibit similar shapes, indicating that the saline electrolyte has negligible effect on their electrical performance. A prominent decrease in the semicircle diameter for the PNE is observed, which corresponds to a decreased charge-transfer resistance due to the increased PNE surface area, in turn resulting in improved LFP signals. In order to check the reproducibility of EIS results, we analyzed two more PNE and CNE samples (N = 2, N = 3) (Figure S3), and the Nyquist plot and Bode plot quite clearly confirms PNE have better charge-transfer resistance as compared with CNE.

**Figure 5:**
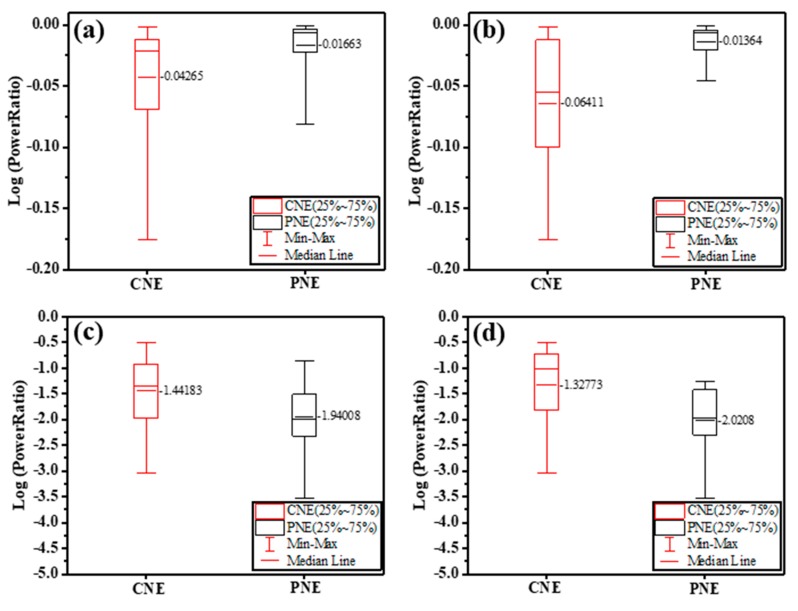
LFP signals measured from the cortices of Sprague-Dawley rats in the range 1 Hz to 49 Hz at (**a**) 1 mm to 5 mm (1 mm increment) and (**b**) specifically at 1 mm depth; Noise recorded in the range 59 Hz to 61 Hz (**c**) at 1 mm to 5 mm (1 mm increment), and (**d**) specifically at 1 mm depth.

LFP was recorded from the cortices with CNE and PNE. Since LFPs are commonly measured at frequencies below 50 Hz [37,38], log power ratios of the measured 1 to 49 Hz signals were considered as the LFP signal. Between the electrodes, the measured 1 to 49 Hz signal intensities showed a significant difference (F (5,42) = 14, *p* < 0.001). The CNE signals showed significantly lower amplitudes than those from the PNE (*p* < 0.05), as seen in Figure 5a. Measured 1 to 49 Hz signals recorded at 1 mm depth showed a significant difference between CNE and PNE, with the CNE showing enhanced LFP signals (F (5,42) = 7.86, *p* < 0.001), as seen in Figure 5b.

Noise, taken as the log power ratio of the signals from 59 Hz–61 Hz, of CNE and PNE showed higher values for CNE in pooled data for all depths (From 1 mm to 5 mm depth, at 1 mm increment) (F (5,42) = 17.96, *p* < 0.001), see Figure 5c. Noise recorded at specifically 1 mm depth exhibited a significant difference among the neural electrodes (F (5,42) = 10.16, *p* < 0.001) where CNE significantly has higher noise than PNE (Figure 5d).The power spectrum data is given in Figure S4, supplementary information. 

In the present study, LFP signal intensities were measured from the cortices of Sprague-Dawley rats, at increments of 1 mm depth, over the frequency range 1 Hz–49 Hz and 59 Hz–61 Hz. In comparison to conventional smooth-surfaced neural electrodes, porous neural electrodes (PNEs) exhibit reduced noise that, in turn, enhances LFP signal detection. We believe the key factor contributing to the enhanced PNE LFP signal is reduced noise which originates with the higher PNE surface area that, in turn, leads to lower charge transfer resistance.

## 4. Conclusions

In the current research article, we developed a neural electrode possessing a nano/micro-scale porous surface topology for improved LFP measurement. The traditional silicon based electrodes/arrays were commonly used in many previous reports. But, here, we developed the single brain electrode with easy synthetic procedure and cost effective approach. The synthesis was carried out with simple anodization technique of conventional smooth-surface neural electrodes, which resulted in the needle possessing a nano/micro-porous topology of significantly increased surface area. A significantly lower charge transfer resistance for the PNE was observed from impedance spectroscopy measurements. Both CNEs and PNEs were applied for measurement of local field potential using Sprague-Dawley rats; the PNEs showed significantly enhanced LFP signal with lower noise. At present, we have only tested single PNEs for LFP detection, but with encouraging results obtained, we would test multiple arrays as well in our future studies. We believe the PNEs offer researchers an exciting new tool for neural network research. As compared with conventional brain electrode, the modified surface topology of as developed porous electrode demonstrate higher measured amplitudes with lower noise levels. Therefore, we believe that our current research opens up new challenges for future research in the field of LFP measurement.

## Supplementary Materials

The following are available online at http://www.mdpi.com/1996-1944/12/3/542/s1, Figure S1: Surface FE-SEM images of second set (**a**) CNE and (**b**) PNE, (**c**) magnified image of (**b**), and (**d**) cross-sectional image of PNE. The insets of (**a**,**b**) show needle tips, Figure S2: Surface FE-SEM images of third set (**a**) CNE and (**b**) PNE, (**c**) magnified image of (**b**), and (**d**) cross-sectional image of PNE. The insets of (**a**,**b**) show needle tips, Figure S3: Electrochemical impedance spectroscopy data: (**a**,**b**) Nyquist plot and (**c**,**d**) Bode plot of second (N = 2) and third set (N = 3) of CNE and PNE, Figure S4: Power spectrum data of (**a**) CNE, and (**b**) PNE, Table S1: EDS (Energy Dispersive Spectrometer) data before and after anodization (second set of PNE and CNE), Table S2: EDS (Energy Dispersive Spectrometer) data before and after anodization (third set of PNE and CNE).
