# Supplementary material for: Novel Porous Brain Electrodes for Augmented Local Field Potential Signal Detection
Source: Materials (Basel). 2019 Feb 12;12(3):542. doi: 10.3390/ma12030542 (PMC6384777; doi:10.3390/ma12030542)
Supplement: Supplementary file 1 [file materials-12-00542-s001.pdf]

# Supplementary Information

## Novel Porous Brain Electrodes for Augmented Local Field Potential Signal Detection

**Sung Hyun Lee, Kyeong-Seok Lee, Saurav Sorcar, Abdul Razzaq, Maan-Gee Lee and Su-Il In**

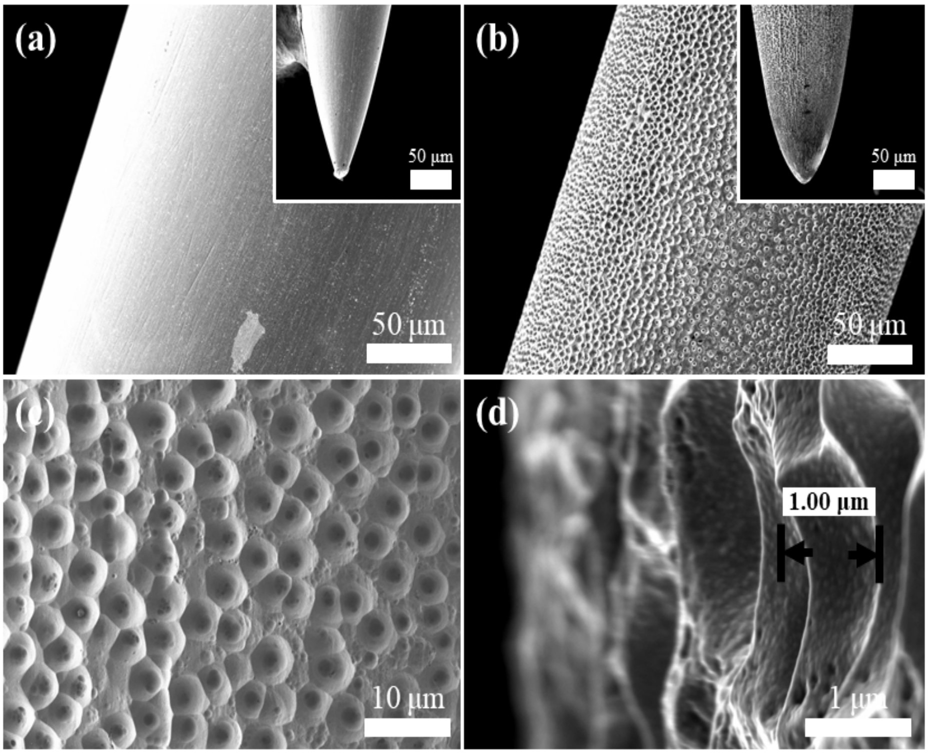


**Figure 1.** Surface FE-SEM images of second set (**a**) CNE and (**b**) PNE, (**c**) magnified image of (**b**), and (**d**) cross-sectional image of PNE. The insets of (**a**,**b**) show needle tips.

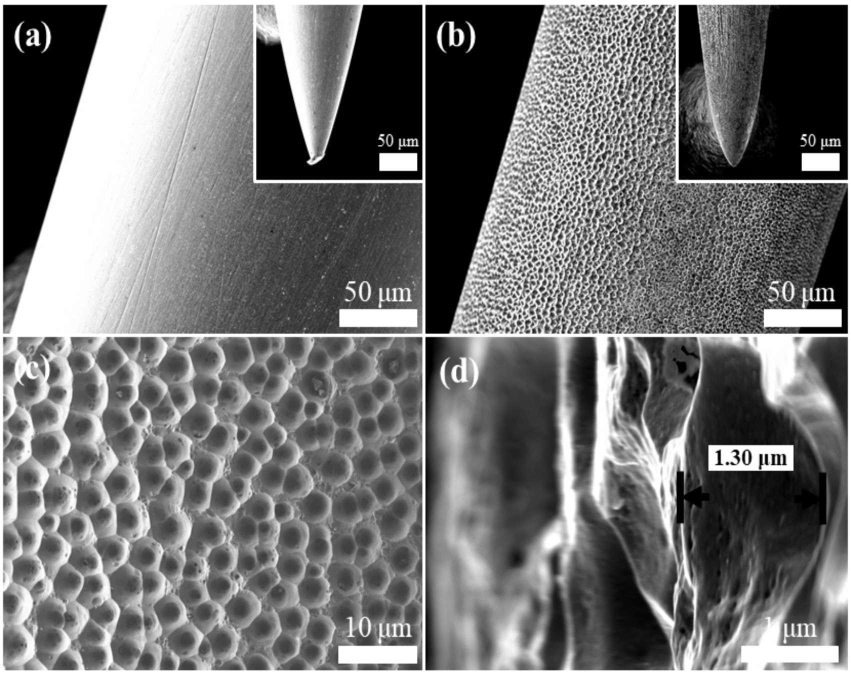


**Figure S2.** Surface FE-SEM images of third set (**a**) CNE and (**b**) PNE, (**c**) magnified image of (**b**), and (**d**) cross-sectional image of PNE. The insets of (**a**,**b**) show needle tips.

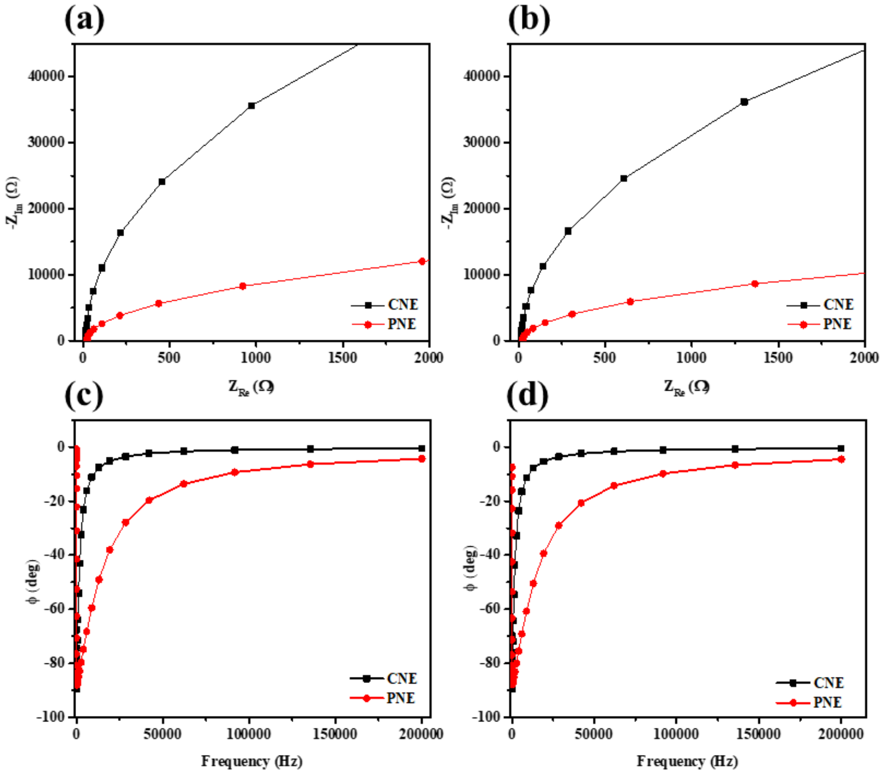


**Figure S3.** Electrochemical impedance spectroscopy data: (**a**,**b**) Nyquist plot and (**c**,**d**) Bode plot of second (N = 2) and third set (N = 3) of CNE and PNE.

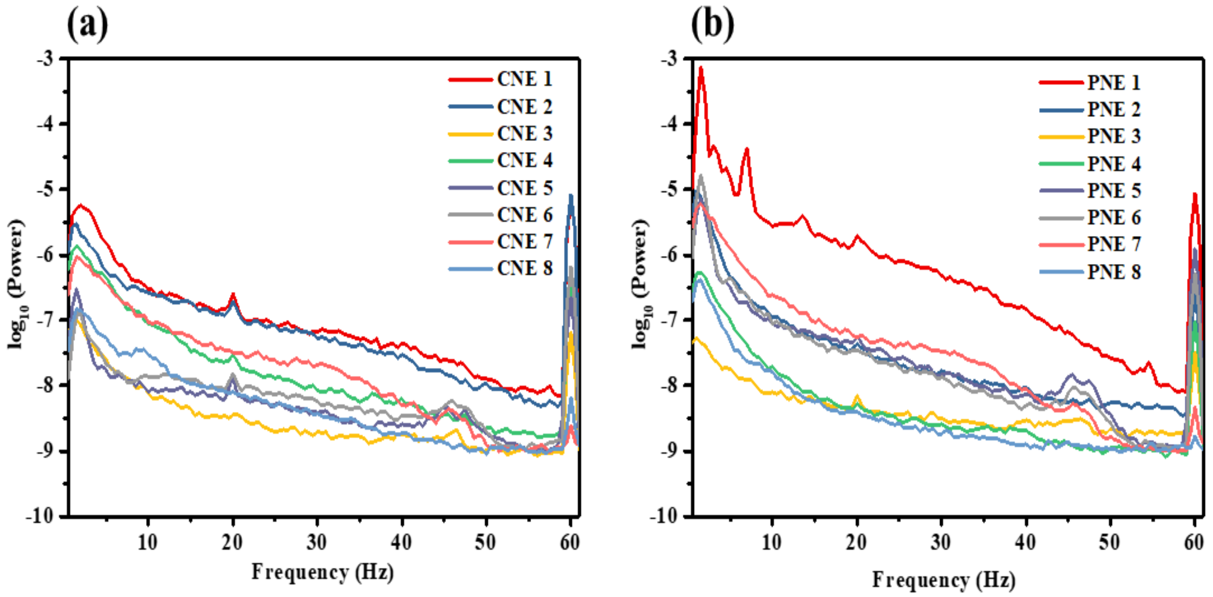


**Figure S4.** Power spectrum data of (**a**) CNE, and (**b**) PNE.

**Table S1.** EDS (Energy Dispersive Spectrometer) data before and after anodization. (second set of PNE and CNE).

| Elements | Before Anodization (CNE) | | After Anodization (PNE) | |
|---|---|---|---|---|
| | Atom. C (at. %) | Error (%) | Atom. C (at. %) | Error (%) |
| Fe | 62.12 | 2.10 | 61.24 | 1.99 |
| Cr | 17.96 | 0.58 | 18.62 | 0.58 |
| C | 12.99 | 0.58 | 14.15 | 0.60 |
| Ni | 6.82 | 0.30 | 5.88 | 0.25 |
| Al | 0.10 | 0.03 | 0.11 | 0.03 |

**Table S2.** EDS (Energy Dispersive Spectrometer) data before and after anodization. (third set of PNE and CNE).

| Elements | Before Anodization (CNE) | | After Anodization (PNE) | |
|---|---|---|---|---|
| | Atom. C (at. %) | Error (%) | Atom. C (at. %) | Error (%) |
| Fe | 61.77 | 2.07 | 62.19 | 2.10 |
| Cr | 18.49 | 0.59 | 18.23 | 0.59 |
| C | 13.17 | 0.59 | 12.99 | 0.58 |
| Ni | 6.50 | 0.28 | 6.52 | 0.29 |
| Al | 0.07 | 0.03 | 0.06 | 0.03 |
